# Identification of *Borrelia* protein candidates in mouse skin for potential diagnosis of disseminated Lyme borreliosis

**DOI:** 10.1038/s41598-017-16749-9

**Published:** 2017-12-01

**Authors:** Antoine Grillon, Benoît Westermann, Paola Cantero, Benoît Jaulhac, Maarten J. Voordouw, Delphine Kapps, Elody Collin, Cathy Barthel, Laurence Ehret-Sabatier, Nathalie Boulanger

**Affiliations:** 1 EA7290, Virulence bactérienne précoce, groupe Borréliose de Lyme, Facultés de Médecine et de Pharmacie, Fédération de Médecine Translationnelle de Strasbourg, Université de Strasbourg, F-67000 Strasbourg, France; 2 Laboratoire de Spectrométrie de Masse BioOrganique, Université de Strasbourg, CNRS, IPHC UMR 7178, F-67000 Strasbourg, France; 3 Laboratory of Ecology and Evolution of Parasites, Institute of Biology, University of Neuchâtel, Neuchâtel, Switzerland

## Abstract

In vector-borne diseases, the skin plays an essential role in the transmission of vector-borne pathogens between the vertebrate host and blood-feeding arthropods and in pathogen persistence. *Borrelia burgdorferi* sensu lato is a tick-borne bacterium that causes Lyme borreliosis (LB) in humans. This pathogen may establish a long-lasting infection in its natural vertebrate host where it can persist in the skin and some other organs. Using a mouse model, we demonstrate that *Borrelia* targets the skin regardless of the route of inoculation, and can persist there at low densities that are difficult to detect via qPCR, but that were infective for blood-feeding ticks. Application of immunosuppressive dermocorticoids at 40 days post-infection (PI) significantly enhanced the *Borrelia* population size in the mouse skin. We used non-targeted (Ge-LC-MS/MS) and targeted (SRM-MS) proteomics to detect several *Borrelia*-specific proteins in the mouse skin at 40 days PI. Detected *Borrelia* proteins included flagellin, VlsE and GAPDH. An important problem in LB is the lack of diagnosis methods capable of detecting active infection in humans suffering from disseminated LB. The identification of *Borrelia* proteins in skin biopsies may provide new approaches for assessing active infection in disseminated manifestations.

## Introduction

Lyme borreliosis (LB) is the most common vector-borne disease in the Northern Hemisphere^1^. The spirochete bacteria that cause LB, *Borrelia burgdorferi* sensu lato (sl), are transmitted by hard ticks belonging to the genus *Ixodes* ^2^. In Europe, the three most frequent LB pathogens are *B. burgdorferi* sensu stricto (ss), *B. afzelii* and *B. garinii*. In North America, *B. burgdorferi* sensu stricto causes the vast majority of Lyme disease cases with just a few cases caused by *B. mayonii* ^3^ *. Ixodes* ticks have three blood-feeding stages: larva, nymph, and adult. The larval and nymph ticks acquire *Borrelia* pathogens from infected hosts and subsequently develop into infected nymphs and female respectively, that transmit the pathogen to naive hosts the following year^4^.

The skin of the vertebrate host plays a critical role in the biology of LB. *Ixodes* ticks use their mouthparts to cut through the skin of their vertebrate host, and blood feeding can take 3 to 10 days depending on the stage^5^. During the blood meal, *Borreliae* migrate from the tick midgut to the tick salivary glands^6^ and are inoculated with the tick saliva into the skin of the vertebrate host^7^. At the site of the tick bite, *Borrelia* pathogens multiply in the skin with a peak abundance at 5 to 15 days^8,9^. They subsequently migrate through the extracellular matrix, enter capillaries, and disseminate to distant tissues^10^. In competent reservoir hosts, *Borrelia* pathogens establish a chronic infection in the skin and other organs such as the heart, bladder, and joints^11^. A study on the kinetics of infection with *B. burgdorferi* ss in laboratory mice showed that live spirochetes can be cultured from mouse skin up to one year post-infection^11^. Transmission of *Borrelia* from infected rodent hosts to feeding *Ixodes* ticks is highly efficient over the duration of the infection^12,13^. Studies on *B. afzelii* in wild and laboratory rodents have shown that the spirochete density in the skin determines the success of host-to-tick transmission^14–16^. Taken together, these observations suggest that the skin of the vertebrate reservoir host plays a critical role in receiving, hosting, and transmitting the *Borrelia* pathogens.

The skin also plays an important role in human LB. The migration of the spirochetes through the human skin is believed to cause the most reliable diagnostic symptom of early LB, the erythema migrans, which is typically an expanding rash that occurs shortly after the infected tick bite^1^. Patients with late LB can suffer from a skin disorder known as acrodermatitis chronica atrophicans. Other symptoms of disseminated LB include arthritis, and neurological problems^1^. There is currently much interest in the processes that occur in disseminated and late LB and in the mechanisms that allow the spirochetes to persist or not in the vertebrate host^17^. In this context, the role of the skin in the physiopathology of LB remains poorly investigated. Moreover, little is known about how *Borreliae* are acquired by naïve ticks. Spirochetes present in the host skin are either passively acquired during the tick blood meal or they actively migrate to the tick mouthparts in response to chemo-attractants^18,19^.

To better understand the role of the skin and of tick saliva in LB and more specifically in disseminated infections, we inoculated *Borrelia* in laboratory mice by three different routes: (1) intradermal syringe inoculation, (2) intraperitoneal syringe inoculation, and (3) infected ticks. We studied whether feeding the mice by naïve ticks and the immunosuppressive effects of tick saliva enhanced the abundance of spirochetes in the host skin. To also demonstrate the presence of live *Borreliae* in the skin, we applied an immunosuppressive dermocorticoid. This approach allowed us to detect an increase in the number of bacteria in the skin as shown by qPCR. To identify bacterial proteins in the mouse skin, we conducted a proteomic analysis using gel electrophoresis followed by liquid chromatography and tandem mass spectrometry (Ge-LC-MS/MS). We have recently shown that this method is efficient at detecting *Borrelia* in mouse skin shortly after tick-to-host transmission^20^. Detection of *Borrelia* proteins in the skin is of great interest as a potential diagnostic tool of active infection in patients with disseminated and late LB.

The diagnosis of LB is often difficult, especially at the late stages of the disease, due to the variety of clinical symptoms. Currently available diagnostic tests include serology and detection of spirochetes in tissue biopsies using PCR and/or culture^21^. Limitations exist for both of these diagnostic approaches. The sensitivity of serological methods ranges from 50 to 99% depending on the stage of the disease^22^. Furthermore, serology can indicate exposure to the pathogen but cannot prove the existence of an active infection. With respect to tissue biopsies in late LB, the sensitivity of the PCR can vary greatly depending on the type of tissue. For example, in European patients with untreated Lyme borreliosis, the sensitivity of PCR is high for ACA (50–70%) and synovial fluid (50%) and much lower in cerebrospinal fluid (15–30%) and rarely positive in serum^23^. New diagnostic approaches are therefore required to prove active infection in individuals with late or chronic LB. One promising diagnostic method is selected reaction monitoring mass spectrometry (SRM-MS). This mass spectrometry-based technique is highly efficient for biomarker identification and validation in a diversity of biological fluids such as blood, plasma, and urine^24–31^. As the skin is an organ where *Borrelia* persists in mice^32^ and dogs^33^, we used a mouse model to test skin tissue to identify *Borrelia* proteins as markers of disseminated infection. These proteins could be potential candidates for the diagnosis of disseminated and late LB in humans.

## Results

### *B. burgdorferi* ss N40 disseminates and infects the mouse skin regardless of the mode of inoculation

To demonstrate the role of the skin as a homing and persistence organ for *Borrelia* pathogens in disseminated infections, mice were infected with *B. burgdorferi* ss strain N40 using two inoculation routes: intradermal and intraperitoneal. We selected the time point of 40 days post-infection (PI), because previous works have shown that all distant organs are positive at day 15 PI^11,34^. Different mouse tissues (blood, heart, joints, and ear for distant skin) were tested for the presence of *Borrelia* using culture. All tissue biopsies that gave spirochete-negative cultures were re-tested using PCR targeting the *flagellin* gene (Table 1). In the disseminated phase of the disease (40 days PI), the heart, joints, and ears were the sites of spirochete persistence, whereas the blood cultures were negative (Table 1). The results were the same regardless of the route of inoculation (p > 0.05). Interestingly, despite being bypassed by the intraperitoneal inoculation, the ear skin was strongly positive for *Borrelia* after dissemination. This result demonstrates that strain N40 of *B. burgdorferi* ss establishes a persistent infection in the skin after its dissemination to the distant organs in our C3H/HeN mouse model as shown previously^11^.

**Table 1:** *Borrelia burgdorferi* ss N40 is present in organs but not in blood of C3H/HeN mice at 40 days after intradermal or intraperitoneal inoculation. *Borrelia* spirochetes were detected by culturing sub-samples of tissue biopsies in BSK media. Tissue biopsies that tested negative using the culture method were re-tested using qPCR.

Inoculation route	Mouse organ tested				
	Blood	Heart	Joint	Ear skin	Serology
Intradermal	0% (0/13)	62% (8/13)	92% (12/13)	92% (12/13)	100% (13/13)
Intraperitoneal	0% (0/9)	66% (6/9)	100% (9/9)	100% (9/9)	100% (9/9)

### Live *Borrelia burgdorferi* ss N40 can achieve transmission from the mouse skin to naïve *Ixodes ricinus* nymphs during disseminated infection

To demonstrate the ability of persistent bacteria in the skin to infect ticks, we fed naïve *I. ricinus* nymphs on mice previously infected via intradermal or intraperitoneal inoculation with *B. burgdorferi* ss strain N40. Interestingly, mouse-to-tick transmission of the bacteria occurred as early as 5–8 hours following nymphal infestation (Table 2). For the mice in the intradermal group, nymphs acquired *B. burgdorferi* ss strain N40 at high efficiency (80.0%) at 8 hours following attachment (Table 2). In contrast, for the mice in the intraperitoneal group, nymphs acquired strain N40 at high efficiency (70.0%) at 5 days following attachment (Table 2). These data suggest that efficient mouse-to-tick transmission occurred much earlier when the spirochetes were inoculated directly into the skin.

**Table 2:** The percentage of *Ixodes ricinus* nymphs that acquired *Borrelia burgdorferi* ss N40 increases over the duration of tick feeding on infected mice. Mice had been infected 40 days before the nymphal infestation via either intradermal or intraperitoneal inoculation.

Time of feeding duration	Intradermal		Intraperitoneal	
	Infected ticks/total ticks (%)	Number of mice	Infected ticks/total ticks (%)	Number of mice
5 h	2/9 (22.2)	4 [0–3]*	0/12 (0.0)	5 [1–5]*
8 h	12/15 (80.0)	7 [0–3]	1/16 (6.3)	4 [2–5]
24 h	13/18 (72.2)	7 [1–3]	1/16 (6.3)	5 [2–5]
3 d	14/28 (50.0)	9 [1–3]	1/21 (50.0)	7 [2–5]
5 d	11/11 (100.0)	4 [2–3]	7/10 (70.0)	5 [1–4]
7 d	6/7 (85.7)	4 [1–3]	5/7 (71.4)	5 [1–3]

^*^In brackets, number of ticks attached on mice or, fed and detached from mice.

### Reactivation of *B. burgdorferi* ss N40 in the mouse skin by tick blood feeding

**Figure 1:**
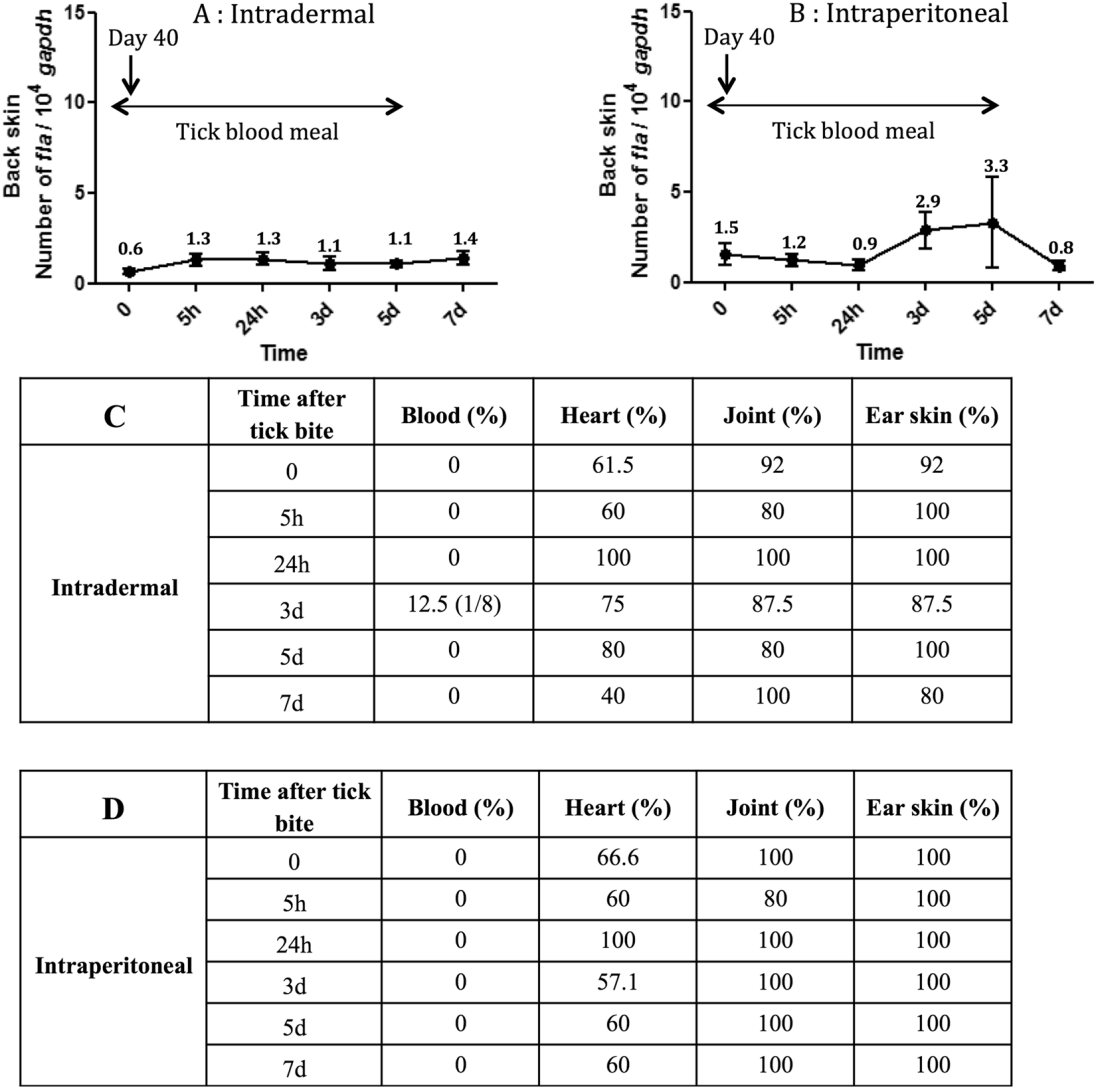
Feeding of uninfected *Ixodes ricinus* nymphs on chronically infected mice does not affect the Borrelia spirochete load in the mouse skin surrounding the tick feeding lesion. Mice were infected with *Borrelia burgdorferi* ss strain N40 via (**A**) intradermal or (**B**) intraperitoneal inoculation. At 40 days post-infection, the mice were infested with 5 to 10 uninfected *I. ricinus* nymphs. At different time points during nymphal attachment (5 h, 24 h, 3d, 5d, 7d), the mice were sacrificed and the mouse skin surrounding the tick feeding lesions was excised. The *Borrelia* load in the mouse skin was estimated using qPCR. Different mouse organs were tested by culture for seven days to determine the presence of *Borrelia*. Tissue biopsies that yield spirochete-negative cultures were retested using qPCR. The blood remained spirochete-negative over the duration of the tick blood meal, with the exception of one mouse at day 3 that had been inoculated intradermally.

The number of bacteria in the skin did not significantly increase after tick feeding at the site of the tick bite in the back of the mouse (Fig. 1A,B), regardless of the route of inoculation. The blood remained spirochete-free during the blood meal of the uninfected nymphs. This observation suggests that feeding by *I. ricinus* nymphs on the mice did not reactivate growth of *B. burgdorferi* ss strain N40 in the skin or the distant organs: heart, joint and ear skin. This result also suggests that the naïve nymphs acquired the spirochetes exclusively from the skin and not from the blood (Fig. 1C,D). The low resident spirochete load was sufficient to ensure efficient transmission to naïve ticks.

### Reactivation of *Borrelia burgdorferi* ss N40 in mouse skin after dermocorticoid application

**Figure 2:**
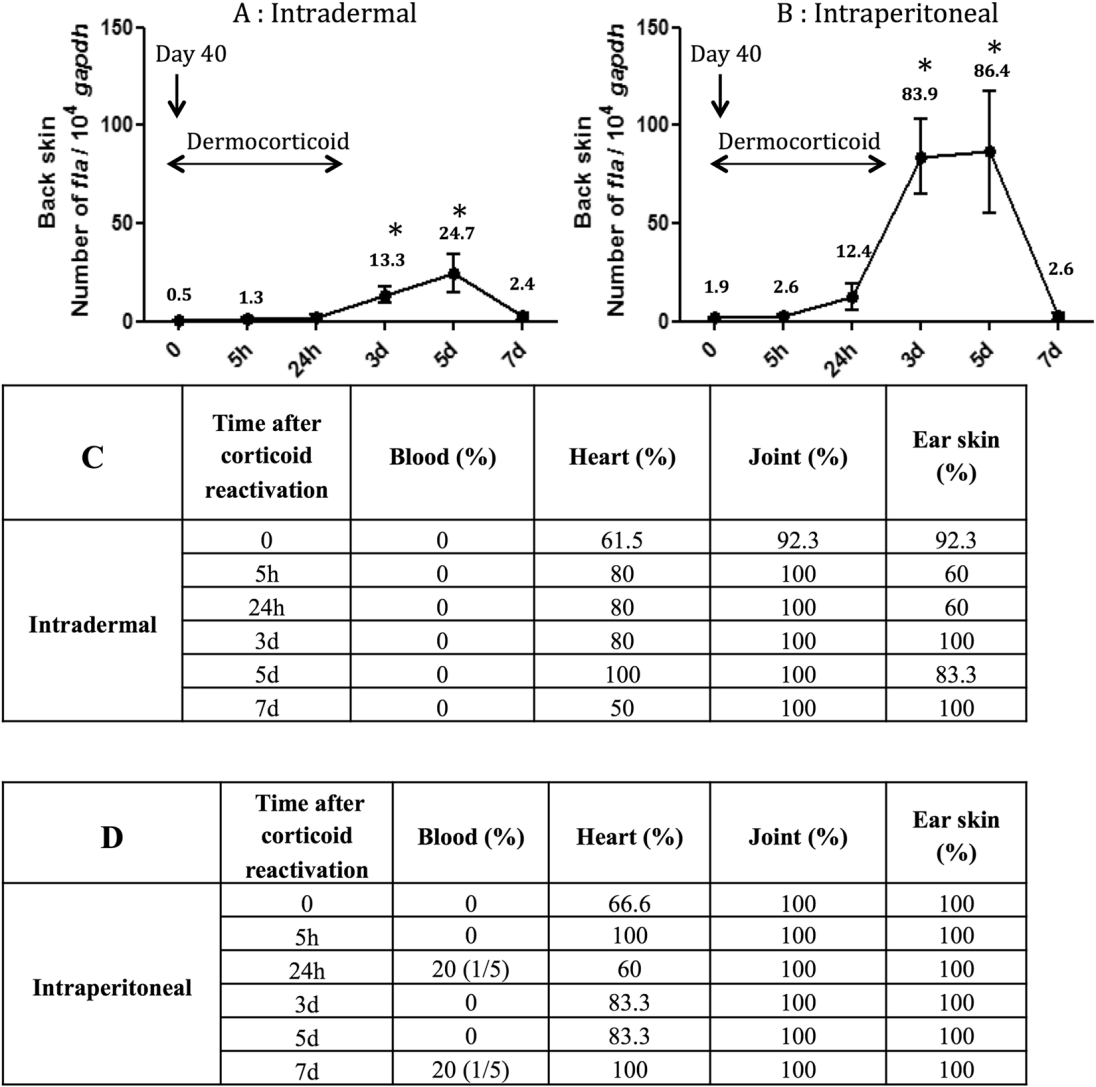
Application of the dermocorticosteroid clobetasol to the skin of chronically infected mice increased the *Borrelia* spirochete abundance in the mouse skin. Mice were infected with *Borrelia burgdorferi* ss strain N40 via (**A**) intradermal or (**B**) intraperitoneal inoculation. At 40 days post-infection, the mice were treated with clobetasol for two days. At different time points following the clobetasol treatment (5 h, 24 h, 3d, 5d, 7d), the mice were sacrificed and the mouse skin where the dermocorticosteroid had been applied was excised. The *Borrelia* load in the mouse skin was estimated using qPCR for seven days. Different mouse organs were tested by culture for seven days to determine the presence of *Borrelia*. Tissue biopsies that yield spirochete-negative cultures were retested using qPCR. The blood remained spirochete-negative over the duration of the tick blood meal, with the exception of two mice in the intraperitoneal group. The asterisk (*) indicates time points where the spirochete load is significantly higher (p < 0.05) than the base line (t = 0).

Corticosteroids administered orally can suppress the immune system and boost *Borrelia* infection^33^. To induce local immunosuppression and demonstrate that *B. burgdorferi* ss N40 can thus multiply in the mouse skin, we applied the dermocorticoid clobetasol to the mouse skin. The clobetasol treatment reactivated spirochete growth and increased the spirochete load in the mouse skin (Fig. 2A,B). In the intradermal group, spirochete load increased significantly at day 3 after the clobetasol treatment, spirochete abundance peaked at day 5, and then decreased to undetectable levels at day 7 (Fig. 2A). A similar pattern was observed in the intraperitoneal group (Fig. 2B). Reactivation was higher in the intraperitoneal group than the intradermal group at day 5 (p = 0.009). The dermocorticoid reactivated the *Borrelia* population in the skin, but the blood remained spirochete-negative in the intradermal group and mostly negative in the intraperitoneal group (Fig. 2C,D). Moreover, of the 81 mouse skin samples tested by PCR (n = 45 for intradermal and n = 36 for intraperitoneal), 92.6% (75/81) had live spirochetes in BSK culture. Altogether, these results demonstrate that after dissemination to distant organs, live *Borrelia* spirochetes persist in the skin of mice and can be reactivated locally by a dermocorticoid.

### Identification of *Borrelia burgdorferi* ss N40 proteins in mouse skin by Ge-LC/MS-MS during disseminated infection after intraperitoneal inoculation

We have shown previously that Ge-LC-MS/MS is an efficient approach to identify *Borrelia* proteins in the skin of infected mice shortly after spirochete transmission and during the peak of spirochete multiplication at day 7^20^. In mice infected intradermally with *B. burgdorferi* ss N40 and not treated with clobetasol, the spirochete density was very low in the skin (≤2 *flagellin*/10^4^ *gapdh*) after disseminated infection. Flagellin was the only *Borrelia* protein identified by Ge-LC/MS-MS with confidence in one of five mice analyzed (data not shown).

To analyze proteins present in the skin of the mice, we selected a sub-sample of six mice for which the density of spirochetes in the skin was high (90 to 259 *fla*/10^4^ *gapdh*). These mice had been inoculated via the intraperitoneal route and treated with clobetasol at 40 days PI to reactivate the *Borrelia* infection in the mouse skin. As expected, we identified mostly mouse proteins (3077 to 3761 different mouse proteins). However, we also detected up to eight *Borrelia* proteins including flagellin, VlsE cassette 2, VlsE cassette 13, DbpA, GAPDH and hypothetical protein BbuN40_0563 (Table 3). *Borrelia* proteins were more likely to be found in mouse tissue samples with high spirochete loads (as estimated by the *fla* qPCR). For example, seven *Borrelia* proteins were detected in the skin of mouse 6, which had the highest spirochete density in this sub-sample (259 *fla*/10^4^ *gapdh*).

**Table 3:** Identification of *Borrelia* proteins by Ge-LC-MS/MS in mouse skin infected with *Borrelia burgdorferi* ss N40.Mice were inoculated intraperitoneally and the *Borrelia* infection was reactivated with clobetasol at 40 days post-infection. Proteins were identified using both Mascot and OMSSA algorithms. The numbers of identified peptides are in brackets.

Mouse ID #	1	2	3	4	5	6
Spirochete density *(fla*/10^4^ *gapdh)*	*119*	*103*	*203*	*90*	*99*	*259*
**Mouse proteins** ^**a**^	**3733**	**3761**	**3723**	**3552**	**3077**	**3373**
Flagellin	X (5)	X (1)	X (5)		X (2)	X (4)
VlsE	X (3)	X (4)	X (6)		X (1)	X (6)
DbpA	X (1)	X (1)				
GAPDH		X (1)				X (1)
BbuN40_0563^b^						X (1)
Lipoprotein gi|256055301						X (1)
Chaperonin GroEL						X (1)
Elongation factor Tu						X (1)

^a^Number of identified mouse proteins.

^b^Hypothetical protein BbuN40_0563.

### Identification of *Borrelia* proteins in mouse skin during disseminated infection after intradermal inoculation by Ge-LC/MS-MS

In the natural life cycle, *Borrelia* is inoculated into the dermis by the tick mouthparts, which cut the host skin. Intradermal inoculation more closely resembles an infected tick bite than intraperitoneal inoculation. We therefore used the intradermal route to infect mice with strains of the three most common LB pathogens in humans: *B. burgdorferi* ss, *B. afzelii* and *B. garinii*. After clobetasol reactivation, we excised the skin surrounding the inoculation site and estimated the spirochete load using qPCR (between 14 and 24 mice were tested for each *Borrelia* species; Table S1 in the supplementary data). The species that multiplied most efficiently in the mouse skin after clobetasol treatment was *B. afzelii* (Table 4 and Table S1 in the supplementary data). Before clobetasol application, the PCR data range between 1.63 and 4.44 (Data not shown). For proteomic studies, we selected the mouse skin biopsies with the highest spirochete loads and some with lower spirochete loads (Table 4). We detected more than 3000 mouse proteins and 3 *Borrelia* proteins: VlsE, flagellin and GAPDH. Compared to *B. afzelii*, the two other *Borrelia* species multiplied less efficiently in the mouse skin.

**Table 4:** Identification of *Borrelia* proteins by Ge-LC-MS/MS in mouse skin infected with one of three different species of *Borrelia burgdorferi* sensu lato.

Mouse ID #	*B. burgdorferi* ss N40			*B. afzelii* NE4049							*B. garinii* PBi		
	1	2	3	1	2	3	4	5	6	7	1	2	3
Spirochete density *fla*/10^4^ *gapdh*	*24*	*10*	*43*	*304*	*151*	*3*	*69*	*92*	*38*	*112*	14	30	19
**Mouse proteins**	**3302**	**3458**	**3482**	**3440**	**3147**	**3580**	**3513**	**3009**	**2979**	**3077**	**3381**	**3374**	**3279**
Flagellin				X (4)			X (3)						
VlsE			X (3)	X (1)				X (1)		X (1)			
GAPDH				X (2)									

Mice were inoculated intradermally and the *Borrelia* infection was reactivated with clobetasol at 40 days post-infection. Proteins were identified using both Mascot and OMSSA algorithms. The numbers of identified peptides are in brackets.

### Identification of *Borrelia afzelii* proteins in mouse skin after infectious tick bite

We have previously shown that infected nymphs inoculate a very small number of spirochetes into the rodent host during the blood meal^9^. Since *B. afzelii* multiplies more efficiently than the two other *Borrelia* species, we used this species to analyse *Borrelia* proteins present in the mouse skin after a tick bite. We infected mice with 10 *B. afzelii*-infected *I. ricinus* nymphs (mean of 80% of infected nymphs) and *Borrelia*e were reactivated with clobetasol at 40 days post-infection to ensure an efficient detection of bacterial proteins in the skin. After PCR quantification, the 4 mice presented a very high density of *fla*/*gapdh*. The detection of a panel of *Borrelia* proteins in the skin samples of these mice was very good since 36 *Borrelia* proteins were identified, and 10 with at least 3 different peptides (Table 5 **)**. Three *Borrelia* proteins were also recovered from the mice that had been infected via needle inoculation: flagellin, VlsE and GAPDH. Seven other *Borrelia* proteins were unique to the mice infected via tick bite: flagellar filament outer layer, L-lactate dehydrogenase, enolase, oligopeptide ABC transporter, neutrophil activating protein, and 2,3-biphosphoglycerate-dependent phosphoglycerate mutase.

**Table 5:** Identification of *Borrelia* proteins by Ge-LC-MS/MS in mouse skin infected with *Borrelia afzelii* isolate NE4049.

Mouse ID #	1	2	3	4
Spirochete density *fla*/10^4^ *gapdh*	*876*	*602*	*1231*	*723*
**Mouse proteins**	**4489**	**4156**	**3961**	**4372**
Flagellin	X (11)	X (8)	X (8)	X (10)
Flagellar filament outer layer	X (1)	X (4)	X (1)	X (6)
VlsE	X (3)	X (2)	X (2)	X (2)
L-lactate dehydrogenase	X (3)	X (5)		X (4)
GAPDH	X (4)	X (5)	X (7)	X (6)
Enolase	X (3)	X (1)		X (2)
Oligopeptide ABC transporter gi|111115153	X (3)	X (2)	X (1)	X (6)
Neutrophil activating protein	X (3)	X (1)		
2,3-biphosphoglycerate-dependent phosphoglycerate mutase	X (2)	X (1)	X (2)	X (3)
Elongation factor Tu	X (3)	X (7)	X (2)	X (9)

Mice were infected via tick bite (10 nymphs) and the *Borrelia* infection was reactivated with clobetasol at 40 days post-infection. Proteins were identified using both Mascot and OMSSA algorithms. The numbers of identified peptides are in brackets. Of the 36 proteins identified, only those identified with at least 3 different peptides in at least one biopsy are listed.

We also analyzed mouse skin biopsies after infesting each mouse with 5 *B. afzelii*-infected nymphs (mean of 80% of infected nymphs). The skin infection rate was lower (Supplementary data – Table 2) and no proteins were identified using both Mascot and OMSSA algorithms. Some proteins were identified by a single algorithm only (data not shown) but were not different from the ones identified previously.

### Detection of specific *Borrelia* proteins in infected mouse skin by targeted proteomics (Ge-LC-SRM)

We used targeted proteomics to improve detection and quantification of *Borrelia* proteins in mouse skin. Among all *Borrelia* proteins identified by Ge-LC-MS/MS analyses, 14 proteins were selected for a targeted Ge-LC-SRM assay. The method monitored 544 transitions corresponding to 67 peptides. To consider sequence variation in *Borrelia* proteins, the selection of proteotypic peptides included a large selection of protein variants, especially for flagellin and VlsE. The specificity and sensitivity of Ge-LC-SRM allowed the detection of *Borrelia* proteins in mouse skin biopsies with lower spirochete densities than by Ge-LC-MS/MS. To assess our Ge-LC-SRM detection method, we examined two mouse skin biopsies with high spirochete densities of either *B. burgdorferi* ss N40 (mouse 1: 259 *fla*/10^4^ *gapdh*) or *B. afzelii* NE4049 (mouse 5: 304 *fla*/10^4^ *gapdh*). After extraction, 75 µg of protein extract was loaded onto the gel (16 bands) and analysed by LC-SRM. The specific detection of each peptide was validated as we observed (1) the co-elution for all transitions, (2) the co-elution between heavy labelled and the endogenous peptide, (3) a consistent ratio between peptide transitions for endogenous and heavy labelled peptides, and (4) a signal greater than 3 times the signal to noise ratio. Several *Borrelia* proteins/peptides were identified in both two biopsies using Ge-LC-SRM (Table 6). This result proves the feasibility of using LC-SRM to detect specific *Borrelia* proteins in infected mouse skin at the disseminated stage of LB (40 days post-infection).

**Table 6:** Targeted detection of bacterial proteins by LC-SRM in mouse skin infected with *Borrelia burgdorferi* ss N40 or *Borrelia afzelii* NE4049.

Detected proteins		*B. burgdorferi* ss N40				*B. afzelii* NE4049			
	Infection Mode	IP	ID	ID	ID	ID	ID	ID	ID
	Spirochete density *fla/ 10* ^*4*^ *gapdh*	*259*	*43*	*15*	*10*	*304*	*69*	*10*	*6*
	Sequence of SRM-monitored peptides	Mouse 1	Mouse 2	Mouse 3	Mouse 4	Mouse 5	Mouse 6	Mouse 7	Mouse 8
DbpA	DITDEIDAIK	N.d.	N.d.	N.d.	N.d.	N.d.	N.d.	N.d.	N.d.
	GVNFDAFK	N.d.	N.d.	N.d.	N.d.	N.d.	N.d.	N.d.	N.d.
	TTANGIIEIVK	N.d.	N.d.	N.d.	N.d.	N.d.	N.d.	N.d.	N.d.
	TVTDAAEQHPTTTAEGILEIAK	**D**	N.d.	N.d.	N.d.	N.d.	N.d.	N.d.	N.d.
	VSENSFILEAK	**D**	N.d.	N.d.	N.d.	N.d.	N.d.	N.d.	N.d.
Flagellin	ANLGAFQNR	**Q (45)**	**Q (5)**	N.d.	N.d.	**Q (36)**	**Q (4)**	N.d.	N.d.
	AINFIQTTEGNLNEVEK	**Q (94)**	N.d.	N.d.	N.d.	**Q (52)**	N.d.	N.d.	N.d.
	INTPASLSGSQASWTLR	**Q (88)**	**Q (14)**	N.d.	N.d.	**Q (77)**	N.d.	N.d.	N.d.
	ASDDAAGMGVSGK	**D**	**D**	N.d.	N.d.	**D**	N.d.	N.d.	N.d.
	ELAVQSGNGTYSDSDR	N.d.	N.d.	N.d.	N.d.	N.d.	N.d.	N.d.	N.d.
	GSIQIEIEQLTDEINR	N.d.	N.d.	N.d.	N.d.	N.d.	N.d.	N.d.	N.d.
	IADQAQYNQMHMLSNK	**D**	**D**	N.d.	N.d.	**D**	N.d.	N.d.	N.d.
	MIINHNTSAINASR	**D**	**D**	N.d.	N.d.	**D**	N.d.	N.d.	N.d.
	NNAINAANLSK	N.d.	N.d.	N.d.	N.d.	**D**	**D**	N.d.	N.d.
	NNGINAANLSK	**D**	N.d.	N.d.	N.d.	N.d.	N.d.	N.d.	N.d.
	NSTEYAIENLK	N.d.	N.d.	N.d.	N.d.	**D**	N.d.	N.d.	N.d.
	TAEELGMQPAK	**D**	**D**	N.d.	N.d.	**D**	N.d.	N.d.	N.d.
HP BAPKO_0593	LPLALNLAVSR	N.d.	N.d.	N.d.	N.d.	**D**	N.d.	N.d.	N.d.
Lipoprotein gi|365823350	EFFDWLSK	**D**	N.d.	N.d.	N.d.	N.d.	N.d.	N.d.	N.d.
	GEALSLFFQK	**D**	N.d.	N.d.	N.d.	N.d.	N.d.	N.d.	N.d.
	SLTEIDSGNGIPLVVSDVVK	N.d.	N.d.	N.d.	N.d.	N.d.	N.d.	N.d.	N.d.
	VLTESESNNELK	**D**	N.d.	N.d.	N.d.	N.d.	N.d.	N.d.	N.d.
Vls13	AAAAVSSVSGEQILK	N.d.	N.d.	N.d.	N.d.	N.d.	N.d.	N.d.	N.d.
	AVSSVSGEQILK	N.d.	N.d.	N.d.	N.d.	N.d.	N.d.	N.d.	N.d.
	GIVDAAGTAAGK	**D**	**D**	**D**	N.d.	**D**	N.d.	N.d.	N.d.
	GIVDAAGTAAGKK	**D**	**D**	N.d.	N.d.	N.d.	N.d.	N.d.	N.d.
	IGESADNGAAADADSVK	N.d.	N.d.	N.d.	N.d.	N.d.	N.d.	N.d.	N.d.
	VAAALVLR	**D**	**D**	N.d.	N.d.	**D**	N.d.	N.d.	N.d.
Vls2	AAEAVSSVSGEQILK	**D**	**D**	N.d.	N.d.	N.d.	N.d.	N.d.	N.d.
	AAEEAIVGATGDGTK	**D**	**D**	N.d.	N.d.	N.d.	N.d.	N.d.	N.d.
	*Borrelia* species typing	YES	YES	YES	NO	YES	YES	NO	NO

Growth of spirochetes was reactivated with clobetasol at 40 days after intraperitonal (IP) or intradermal (ID) inoculation. N.d. = Not detected, D = Detected, Q = Detected and quantified. Quantifications are in brackets (fmol/mg skin).

We further assessed the sensitivity of our method by studying the skin biopsies of six additional mice with lower spirochete densities: three infected with *B. burgdorferi* ss N40 (mice 2, 3, and 4 in Table 6) and three with *B. afzelii* NE4049 (mice 6, 7, 8 in Table 6). These biopsies were analysed with the same Ge-LC-SRM approach (50 µg loaded onto the gel, 11 bands). After analysis, we detected *Borrelia* flagellin and VlsE proteins in 3 of these 6 biopsies. By using AQUA® peptide, we estimated the quantity of flagellin protein in two biopsies. A VlsE peptide (GIVDAAGTAAGK) was even detected in a mouse skin biopsy with a very low spirochete density (mouse 3: 15 *fla*/10^4^ *gapdh*). It is noteworthy that we observed a good correlation between our estimates of flagellin protein content (either absolute quantification or total number of detected peptides) and the spirochete density estimated by PCR. Finally, the detection of proteotypic peptides of each *Borrelia* species/strain allowed us to do bacterial species determination when the initial spirochete density was ≥15 *fla*/10^4^ *gapdh*.

For non targeted proteomics, the biopsy infected with *B. burgdorferi* ss N40, a maximum of 18 peptides corresponding to 4 proteins (flagellin, VlsE, DbpA and lipoprotein gi|365823350) were detected. With targeted proteomics, flagellin was detected in this skin biopsy with 8 targeted peptides, and 4 of these peptides had been previously identified by Ge-LC-MS/MS (Table 3). A similar result was observed with the second skin biopsy infected with *B. afzelii*: 9 flagellin peptides were detected by Ge-LC-SRM, of which 4 peptides had been previously identified by Ge-LC-MS/MS (Table 3). Thus, the targeted LC-SRM method detects more peptides than Ge-LC-MS/MS. By using AQUA® peptides, we estimated the quantity of 3 different flagellin peptides in each biopsy (Table 6 **)**.

### Detection of specific *Borrelia* proteins in infected mouse skin by Ge-LC-SRM after antibiotic treatment

We wanted to determine the persistence or not of *Borrelia* (live bacteria, DNA and proteins) in the skin after antibiotic treatment of infected mice (n = 29) by culture, PCR and Ge-LC-MS/MS. After two days of antibiotic treatment (Day 1), all cultures in BSK-H medium were still positive but spirochete quantification was significantly reduced in the mouse skin by PCR, ranging from 0.067 to 0.158 *flagellin* /10^4^ *gapdh*,(Supplementary data – Table 3). No *Borrelia* proteins were identified by Ge-LC-MS/MS. After 2 days of antibiotic treatment (Day 2), all cultures and*Borrelia* PCR assays were negative (Supplementary data – Table 3). Protein detections were not possible (data not shown).

## Discussion

The goal of this study was to identify *Borrelia* proteins in the skin of mice with disseminated infections and use these proteins as candidate markers for disseminated and late diagnosis of Lyme borreliosis. First, we demonstrated that *Borrelia* establishes a persistent infection in the skin of our mouse model C3H/HeN at 40 days post-infection to mimic the disseminated phase of LB whatever the route of inoculation as previously demonstrated^10,34,35^. Interestingly, the skin appears to be a homing site for *Borrelia*, since the pathogen was found in the skin regardless of whether the mice were infected via intradermal or intraperitoneal inoculation. A previous study had found that live spirochetes of *B. burgdorferi* ss strain N40 could be cultured from the skin of laboratory mice as late at 360 days post-infection^11^. We showed that treatment with an immunosuppressive dermocorticosteroid (clobatesol) greatly enhanced growth of *B. burgdorferi* ss strain N40 in the mouse skin. Previous studies on mice with severe combined immunodeficiency have shown that the acquired immune system plays a critical role in controlling the abundance of *Borrelia* spirochetes in the mouse tissues including the skin^36–39^. The present study shows that the local immune response in the mouse skin plays an important role in controlling the spirochete population.

We also showed that *I. ricinus* nymphs feeding at 40 days post-infection could acquire *B. burgdorferi* ss N40 from infected mice. Previous studies have shown that mouse-to-tick transmission of *Borrelia* pathogens can occur over a duration of months and even years^12,13^. The minimal time necessary for *I. ricinus* nymphs to acquire *B. burgdorferi* ss N40 from infected mice was very fast. Already 5 hours after the beginning of the blood meal, the ticks already fixed were positive by PCR (22%) and the rate of infectivity increased rapidly at 8 hours with 80% of infected ticks, especially after intradermal inoculation. This result was similar to an earlier study on *B. burgdorferi* ss in *I. scapularis* where nymphal ticks acquired spirochetes at 8 hours post-attachment^32^. The fast mouse-to-tick transmission suggests that ticks acquire *Borrelia* spirochetes directly from the skin and not by migration of spirochetes from distant organs. This local transmission hypothesis was further supported by our observation that the mouse blood remained spirochete-negative during mouse-to-tick transmission. We found that efficient mouse-to-tick transmission (≥70%) of *B. burgdorferi* ss N40 to *I. ricinus* nymphs occurred 15 times faster in the intradermal group (8 hours) than in the intraperitoneal group (5 days). A possible explanation is that mouse-to-tick transmission was faster in the intradermal group because the spirochetes were inoculated directly into the skin, which is the key organ for mouse-to-tick transmission. Indeed, after intradermal inoculation or nymph infection, we and others have shown that *Borrelia* multiplies intensively in the skin at day 7 and day 15 respectively, whatever the species and the strain [9,10,36]. It is likely that the *Borrelia* population persisting in the skin is different after intradermal inoculation versus intraperitoneal inoculation. Indeed, the skin operates as a filter for *Borrelia* population, constituted of different clones^16,40^, only some of the clones disseminate to the target organs, “while others persist in the skin”. With IP inoculation, the skin did not have an effect on the *Borrelia* population during the inoculation process.

Tick saliva contains a diversity of molecules with anti-hemostatic, anti-inflammatory and immunomodulatory properties, which facilitates the uptake of the blood meal that is essential for tick survival^41–43^. In the present study, we had originally intended to use tick feeding and tick saliva-induced local immunosuppression to boost the local spirochete population in the mouse skin to enhance the probability of detecting *Borrelia* proteins in the skin. We therefore expected that the immunosuppressive properties of tick salivary gland extract (SGE) would have a similar effect to the clobatesol treatment. However, we found no evidence that feeding by *I. ricinus* nymphs enhanced the density of *B. burgdorferi* ss N40 spirochetes in the mouse skin surrounding the feeding lesion. A number of studies have shown that tick saliva enhances *Borrelia* growth *in vitro* ^44,45^ and abundance and *in vivo* ^46,47^. These studies provide evidence that *Borrelia* spirochetes respond to the tick SGE of the tick vector species with which they have co-evolved. In the present study, we found that feeding by *I. ricinus* nymphs did not enhance the density of *B. burgdorferi* ss N40 in the mouse skin. One explanation is that *B. burgdorferi* ss N40 is not stimulated by the tick SGE of *I. ricinus* nymphs as previously shown by Zeidner *et al*.^46^. Another explanation is that the spirochete population in the infected skin surrounding the tick-feeding lesion is likely to be highly dynamic. Future studies on the dynamics of the spirochete population in the host skin during host-to-tick transmission should investigate other combinations of *Borrelia* species and *Ixodes* ticks.

In the present study, we successfully used clobetasol to locally boost *Borrelia* and to detect protein markers of disseminated infection in the mouse skin. We had previously shown that proteomics can be used to detect *Borrelia* in the skin at 7 days after intradermal inoculation when the bacteria multiply rapidly in the mouse skin^48^. In the present study, the two main *Borrelia* proteins that we detected were flagellin and VlsE. VlsE is a well-known *Borrelia* protein that is expressed during disseminated infection and that helps the spirochete to evade the host antibody response^49,50^. This protein is used in commercial ELISA assays for the diagnosis of disseminated LB^21^. For the proteomics study, the mice had been infected with all three *Borrelia* species via intradermal inoculation followed by dermocorticoid reactivation as well as via tick bite (only *B. afzelii* isolate NE4049) followed by dermocorticoid reactivation. For these mice, we got a large panel of *Borrelia* proteins including flagellin and VlsE, but also L-lactate dehydrogenase, neutrophil activating protein (Nap), enolase, oligopeptide ABC transporter, and Elongation factor Tu. Elongation factor Tu is a highly immunogenic protein which is primarily found in the protoplasmic cylinder of spirochetes and is not on the surface of *B. burgdorferi* ^51^. Nap is one of the main *Borrelia* proteins involved in the pathogenesis of the early stages of Lyme arthritis. This protein orchestrates the recruitment of inflammatory cells into the joint cavity^52^. The role of Nap in disseminated stages of LB is unknown and its presence in the mouse skin was unexpected. Peptide ABC transporters may play a major role in nutrition and virulence of spirochetes^53,54^. Several identified proteins like GroEL, oligopeptide ABC transporter, enolase, and GAPDH are known to be expressed in virulence vesicles in *Borrelia* ^55^.

Of the three *Borrelia* species investigated in the proteomics part of the study, *B. afzelii* NE4049 was found to persist and multiply the best in the skin of the mice. In nature, the different *Borrelia* species are associated with different reservoir hosts: *B. afzelii* with rodents, *B. garinii* with birds, whereas *B. burgdorferi* ss is ubiquitous^56^. This host specificity could explain the efficient persistence and multiplication of some *B. afzelii* strains in mouse skin compared to *B. garinii* strains. Previous studies have shown that *B. afzelii* isolate NE4049 establishes a high spirochete load in the skin of laboratory mice compared to another *B. afzelii* isolate E61^14^ *. B. afzelii* NE4049 also has high mouse-to-tick transmission over a duration of 4 months suggesting that it is highly competent at establishing a persistent infection in laboratory mice^13^. Finally, this *B. afzelii* strain is also highly competent at co-feeding transmission^57,58^ which occurs when spirochetes are transmitted between infected and uninfected ticks that feed in close proximity to each other on the same host at the same time^59^. Co-feeding transmission can occur before the establishment of a systemic infection and probably requires the *Borrelia* pathogen to establish a fast-growing spirochete population at the site of the tick-feeding lesion^59^. In summary, our proteomics approach was most successful at detecting *B. afzelii* in mouse skin because this *Borrelia* species is best adapted to live in rodent reservoir hosts.

As *Borrelia* proteins detected after reactivation in mice are present in disseminated infections, we suggest that these proteins could be interesting markers of disseminated LB in human patients. We have shown that our approach is feasible for the diagnosis of early LB^20^. We are currently validating this approach in more patients and we are comparing its efficiency and sensitivity to PCR detection and *Borrelia* culture (personal communication). The skin remains spirochete-positive for months in mice^11^ and also in dogs^60^. The ability of spirochetes to persist in human skin is not known. However, tick bites remain visible for several months in some patients and skin biopsies could be sampled from the site of the tick bite to perform diagnostics. Alternatively, proteomics might be used on other biological fluids such as synovial fluid or cerebrospinal fluid. In addition, we showed that after only two days of antibiotic treatment of *Borrelia*-infected mice, no live bacteria, DNA or proteins were detected. Diagnosis relying on proteomics could help clinicians to distinguish disseminated stage LB efficiently treated with antibiotics from persistent infections or reinfections.

Late disseminated LB is a real problem in humans^1,61^. In some cases, like acrodermatititis chronica atrophicans, it is well established that the disease is linked to the presence of live bacteria^2^. In other cases, clinical symptoms are linked to an inflammatory process that persists in patients, while the bacteria have been killed by an efficient antibiotic treatment^62^. As proteomics detects proteins indicating the presence of metabolically active organisms, this approach could be used in patients with long-lasting symptoms to check for the real persistence of *Borrelia* in their tissues. Thus, the approach presented in this study might offer new diagnostic tools for disseminated and late LB in humans.

## Materials and Methods

### Animals, Borrelia and *Ixodes ricinus* ticks

Pathogen-free male or female C3H/HeN mice were purchased from Charles River Laboratories. Three different strains belonging to the 3 main *Borrelia* species were used for the infection experiment: *B. burgdorferi* ss strain N40, *B. afzelii* strain NE4049, and *B. garinii* strain PBi. These three LB strains were cultured in BSK-H complete medium (Sigma) at 33 °C and used at passage ≤7 for mouse infections. *Ixodes ricinus* ticks were obtained from the pathogen-free tick colony at the University of Strasbourg (France). Nymphal ticks were used to demonstrate transmission of *Borrelia* from infected mice to ticks.

### Infections of mice with *Borrelia burgdorferi* sl

Mice (3–4 weeks old) were infected via syringe inoculation with each of the three *Borrelia* strains or via tick bite with *B. afzelii* strain NE4049 only (Average infection rate = 80%). For syringe inoculation, mice were inoculated intradermally in the dorsal thoracic area (n = 73 mice) or intraperitoneally (n = 63 mice) with 1000 spirochetes in 100 µL of BSK-H. For infection via tick bite, *I. ricinus* nymphs infected with *B. afzelii* strain NE4049 were generated according to a previously described protocol^14,58^. Each mouse was subsequently infected with 5 or 10 nymphs^63^. After 2 to 3 weeks, the presence of *Borrelia*-specific IgG antibodies in the mouse sera was tested by ELISA^64^.

### Transmission of *B. burgdorferi* ss N40 from infected mice to naïve *I. ricinus* nymphs

Transmission of *B. burgdorferi* ss N40 from infected mice to *I. ricinus* nymphs was studied using xenodiagnosis. For this experiment, 35 and 31 mice were inoculated via the intradermal and intraperitoneal route, respectively. The details of the protocol of transmission of *Borrelia* from infected mice to naive *I. ricinus* nymphs were as follows (see also^63^). Forty days after the initial *Borrelia* inoculation, a plastic capsule was attached to the shaved back of each mouse with wax. The nymphs (5) were placed inside the plastic capsule. At each time point of the time series, the mice were killed and the nymphs removed and counted. In our experience, 40% to 80% of the nymphs in the capsule attach to the mouse. Of the nymphs that attach, ~50% are attached at 8 hours. These engorged nymphs were then tested by PCR to detect *Borrelia*. This PCR protocol amplifies a conservative part of the *Borrelia flagellin* (*fla*) gene^64^. DNA was extracted from the ticks on a MagNA Pure apparatus as described previously^34^.

### Reactivation of *B. burgdorferi* ss N40 in the mouse skin by tick blood meal

Ticks might reactivate *Borrelia* in the skin by their saliva. At the different time points during tick blood meal, mice were sacrificed with Forene® inhalation (isoflurane) and a 1-cm^2^ area of dorsal thoracic skin was removed at the site of the tick feeding. Distant organs (heart, joint and ear skin) and the blood were collected. Dorsal and ear skin and blood were tested by culture for the presence of *Borrelia*. Dorsal skin was tested with quantitative PCR to determine whether the feeding of nymphal ticks reactivated spirochetes in the skin. PCR was used to check for the presence of *Borrelia* in the heart and in the joint as described above. Skin samples with the highest spirochete loads at day 3 or 5 were used for proteomics analyses.

### Reactivation of *B. burgdorferi* ss N40 in the mouse skin by dermocorticoid application

*Borrelia burgdorferi* ss N40 spirochetes were reactivated in mouse skin by applying dermocorticoids. For this experiment we used 32 and 27 mice inoculated via the intradermal and intraperitoneal route, respectively. At ~40 days PI, ~10 mg of Dermoval® (0.05% clobetasol was applied to the dorsal thoracic area of the mice, twice a day for 2 days. To determine the temporal dynamics of spirochete reactivation, mice were sacrificed at different times following clobetasol treatment: 0 h, 5 h, 24 h, 3 d, 5 d and 7 d from the first application. Mice were sacrificed by Forene® inhalation (isoflurane). A 1-cm^2^ area of dorsal thoracic skin was removed for quantitative PCR, at the site of dermocorticoid application. In addition, for each mouse at each time point, four organs were collected: heart, joints, dorsal thoracic skin, and cardiac blood. A minimum of 5 mice was used at each time point (maximum 13). Skin samples with the highest spirochete loads at day 3 or 5 were used for proteomics analyses.

### *Borrelia*-infected animals reactivated with dermocorticoid application then treated with antibiotics

Mice were infected with *B. burgdorferi* ss N40 via intradermal inoculation (n = 29). Forty days post-infection, the mice were treated with clobetasol, twice a day for 2 days to reactivate the spirochetes, as described above. Mice were subsequently treated with antibiotics (ceftriaxone 16 mg/kg) intradermally, twice a day for 5 days^65^. The mice were sacrificed at different times following the end of antibiotic treatment: 1, 2, 3, and 7 days. For each mouse, the skin at the site of intradermal inoculation was collected and cultured for *Borrelia*, and frozen at −80 °C for PCR quantification and proteomics analyses. For controls, infected animals were treated with clobetasol, and subsequently inoculated twice a day for 5 days with saline solution instead of antibiotics.

### Proteomics analysis

For the proteomics part of the study, we used skin samples from the mice from the experiments described above, which had been infected with *B. burgdorferi* ss strain N40 via the intradermal route (n = 3) and the intraperitoneal route (n = 6) and treated with clobetasol. We also inoculated mice via the intradermal route with *B. afzelii* strain NE4049 (n = 7) and with *B. garinii* strain PBi (n = 3). Finally, we infected mice via nymphal tick bite with *B. afzelii* strain NE4049 (n = 4). For infection via tick bite, *I. ricinus* nymphs infected with *B. afzelii* strain NE4049 were created according to a previously described protocol^58^. Each mouse was subsequently infested with 5 or 10 nymphs as described previously^63^. After 2 to 3 weeks, the presence of *Borrelia*-specific IgG antibodies in the mouse sera was tested by ELISA as described previously^64^.

### Culture of *B. burgdorferi* sensu lato from organs or blood

For detection of *B. burgdorferi* sl by culture, the different mouse organs were dissected aseptically. Collected organs and blood (3 drops) were placed in 6 ml of BSK-H medium containing 30 μg of rifampicin (BioRad). The tubes were maintained at 33 °C and examined weekly for the presence of spirochetes by dark-field microscopy as described previously^9^. For each organ and blood sample, the tissue material was divided into two parts: the first part was tested for live *Borrelia* spirochetes using culture, if the culture tested negative, the second part was tested for *Borrelia* DNA using qPCR. For all the skin samples, culture and quantitative PCR were performed.

### Estimation of *Borrelia* load in mouse skin by quantitative PCR

Mouse tissue samples were tested for infection with *B. burgdorferi* using quantitative PCR that targeted the *fla* gene, as described previously^66^. DNA was extracted from the skin of individual mice on a MagNA Pure apparatus. Quantification of the *B. burgdorferi*-specific *fla* gene was performed on a LightCycler system (Roche Diagnostics). Quantification of the mouse-specific *gapdh* gene was performed on an ABI Prism 7500 instrument (Applied Biosystem), using a commercial kit (TaqMan rodent GADPH control reagent; Applied Biosystem). The number of *B. burgdorferi* spirochetes in tissue samples was standardized to 10^4^ *gapdh* gene copies^34^.

### Ge-LC-MS/MS analyses

For non-targeted proteomics, samples (5 mg) of mouse skin biopsies were manually extracted by Laemmli buffer and proteins (50 µg) were pre-fractionated onto 12% SDS-PAGE as described^20^. Gel bands (10 ± 1 bands) of 2 mm were excised manually. After reduction, alkylation and trypsin digestion, the peptides were extracted using 80% ACN and 0.1% HCOOH for 90 min at room temperature. After evaporation, the peptides were suspended in 50 µL of 0.1% HCOOH prior to mass spectrometry analyses. LC-MS/MS analyses were performed on a nanoACQUITY Ultra-Performance-LC system hyphenated to a Q-Exactive Plus mass spectrometer as described^67^. Data analysis was performed as described^20^ against an in-house database containing all protein sequences of *Borrelia* and mouse extracted from NCBInr and UniProtKB-SwissProt, respectively. For the bacteria, four different reference databases were used depending on the strain analysed: *B. burgorferi* ss B31 (1758 entries at May 07, 2013), *B. burgdorferi* ss N40 (1480 entries at January 30, 2015), *B. afzelii* Pko (2180 entries at October 16, 2014), and *B. garinii* Pbi (1410 entries at April 08, 2015). For MS^2^ data, parent and fragment mass tolerance was 5 ppm and 0.07 Da and a maximum of 1 missed cleavage was allowed. The Mascot and OMSSA results were independently loaded into the Proline software (Proline Studio Release 1.0). All spectra leading to an identification exceeding a minimum set threshold (Mascot Ion Score > 25, OMSSA −log(e-value) > 7, peptide length > 6 amino acids) and having a pretty rank as defined by Mascot equal to 1 were kept. Resulting spectra were then filtered to obtain a protein false discovery rate of less than 1%^68^.

### Ge-LC-SRM analyses

For targeted proteomics, protein fractionation and *in gel* digestion was achieved as previously described for Ge-LC-MS/MS analyses with the following modifications. First, two different protein quantities, 100 µg and 75 µg, were electrophoresed onto SDS-PAGE to obtain respectively 16 and 11 bands. Second, after protein digestion, extraction and evaporation, the peptides (average of 6.25 µg of proteins per gel band) were suspended in 6 µL of a mixture of diluted heavy labelled peptides (Thermo Fisher) prior to mass spectrometry.

For each band, the totality of the suspended peptide mixture (6 µL) was injected. For LC-SRM assay, 67 proteotypic peptides were selected for 14 targeted proteins (DbpA, enolase, flagellin, GAPDH, HSP90, BB0081, BBP42, BAPKO0593, BAPKO4515, lipoprotein gi|365823350, VlsE, RNA polymerase, histidine kinase, hypothetical protein gi|500023077) and isotopically labelled equivalent peptides were purchased (64 crude PEPotecs peptides and three high purity AQUA® flagellin peptides). Four transitions per peptide corresponding to the most abundant y or b mono- or doubly charged ions were selected for both endogenous and heavy labelled peptides. For each transition, the collision energy was optimized experimentally by testing six values centred on the reference value. The reference value is calculated using the equation given by the supplier. A total of 544 transitions corresponding to 136 precursors were monitored. For the SRM analyses, 6 µL of a mixture of diluted heavy labelled peptides was added to suspend an average of 6.25 µg peptides (100 µg of proteins/16 gel bands or 75 µg of proteins/11 gel bands).

All separations were carried out on Agilent 1100 Series HPLC system (Agilent Technologies). For each analysis, the sample was loaded into a trapping column ZORBAX 300SB-C18 MicroBore Guard 5 µm, 1.0 × 17 mm (Agilent Technologies) at 50 µL/min with an aqueous solution containing 0.1% HCOOH and 2% ACN (solvent A). After 3 min of trapping, the column was put on-line with a ZORBAX 300SB-C18 3.5 µm, 0.3 × 150 mm column (Agilent Technologies). Peptide elution was performed at 5 µL/min using a gradient from 5–45% solvent B (ACN in 0.1% HCOOH) over 57 min. The isolation width for both Q1 and Q3 was set to 0.7 m/z unit. Time-scheduled SRM method targeted the pairs of isotopically labelled peptides/endogenous peptides in ±3 min retention time windows (6 min in total) within a cycle time of 3000 ms. Mass data collected during LC-SRM were processed with the Skyline open-source software package 3.5.9.

### Statistical analyses

For the different kinetics, generally between 4 and 10 mice were used for each time point. For proteomics analyses, for the different infection protocols, between 10 and 22 mice were inoculated. Statistical analyses between the different groups were made with a non-parametric Kruskal-Wallis test followed by a Dunn’s correction.

### Ethics statement

The protocols carried out in this study were approved by the Comité Régional d’Ethique en Matière d’Expérimentation Animale de Strasbourg (CREMEAS - Committee on the Ethics of Animal Experiments of the University of Strasbourg). Name of the ethics statement /N° CREMEAS 2015062210282757 and APAFIS # 887. The protocols performed on animals follow the European guidelines: “directive 2010/63/EU”.

## Electronic supplementary material

Supplementary data
