# Supplementary material for: Identification of Borrelia protein candidates in mouse skin for potential diagnosis of disseminated Lyme borreliosis
Source: Sci Rep. 2017 Dec 1;7:16719. doi: 10.1038/s41598-017-16749-9 (PMC5711925; doi:10.1038/s41598-017-16749-9)
Supplement: Supplementary file 1 — Supplementary data [file 41598_2017_16749_MOESM1_ESM.pdf]

Identification of *Borrelia* protein candidates in mouse skin for potential diagnosis of disseminated Lyme borreliosis

Antoine Grillon[1], Benoît Westermann[2], Paola Cantero[2], Benoît Jaulhac[1], Maarten J. Voordouw[3], Delphine Kapps[1], Elody Collin[1], Cathy Barthel[1], Laurence Ehret-Sabatier[2] Nathalie Boulanger[1]

Supplementary Information

Table S1. Mice (*Mus musculus* strain C3H/HeN) were infected with one of three *Borrelia* pathogens: *B. burgdorferi* ss strain N40, *B. afzelii* strain NE4049, and *B. garinii* strain PBi via intradermal inoculation. The initial inoculum consisted of 1000 spirochetes in 100 µL of PBS. At 40 days post-infection, the mice were treated with the dermacorticoid clobetasol over a period of two days to reactivate the spirochete population in the skin. Following the clobetasol treatment, the mice were sacrificed and the skin surrounding the clobatesol treatment was removed and tested for the spirochete load using qPCR. The data show the number of *Borrelia flagellin* gene copies relative to 10,000 *Mus musculus gapdh* gene copies. *B. afzelii* strain NE4049 establishes a much higher spirochete load in the mouse skin than the other two *Borrelia* species.
In grey, mouse skins selected for proteomic studies (Ge-LC-MS/MS).

	PCR quantification<br>Number of *flagellin* / 10^4 *gapdh*		
Mouse ID #	*B. burgdorferi* ss N40	*B. afzelii* NE4049	*B. garinii* PBi
1	2.685	13.153	13.756
2	28.97	11.089	30.252
3	8.121	6.187	18.796
4	19.312	3.9	7.483
5	3.687	69.33	1.087
7	4.514	2.633	3.926
8	6.661	151.362	4.333
9	4.472	304.39	2.687
10	9.583	140.273	6.519
11	42.594	23.969	5.853
12	2.428	41.209	nq
13	8.37	33.379	nq
14	10.421	28.386	nq
15	24.659	92.363	
16	6.755	37.562	
17	2.648	15.582	
18	2.309	3.066	
19	76.515	111.469	
20	72.72		
21	17.424		
22	44.545		

23	13.182		
24	21.182		
Average quantification	**18.86**	**60.52**	**9.47**

Supplementary Table 2: Mice (*Mus musculus* strain C3H/HeN) were infested with either 10 or 5 *I. ricinus* nymphs that were infected with *Borrelia afzelii* strain NE4049. A random sample of these nymphs had shown that 80% were infected using PCR. At 40 days post-infection, the mice were treated with the dermocorticoid clobetasol over a period of two days to reactivate the spirochete population in the skin. Following the clobetasol treatment, the mice were sacrificed and the skin surrounding the clobatesol treatment was removed and tested for the spirochete load using qPCR. The data show the number of *Borrelia flagellin* gene copies relative to 10,000 *Mus musculus gaphdh* gene copies. The spirochete load in the mouse skin was much higher for the mice infested with 10 nymphs compared to the mice infested with 5 nymphs.

	**PCR quantification**<br>**Number of *flagellin* / 10^4 *gapdh***	
Mouse N°	**10 ticks**	**5 ticks**
1	876	9.143
2	602	13.653
3	1231	9.551
4	723	<0.05
5		54.338

Supplementary Table 3: Mice (*Mus musculus* strain C3H/HeN) were infected with *B. burgdorferi* ss strain N40 via intradermal inoculation. The initial inoculum consisted of 1000 spirochetes in 100 µL of PBS. At 40 days post-infection, the mice were treated with the dermocorticoid clobetasol over a period of two days to reactivate the spirochete population in the skin. The mice were subsequently treated with the antibiotic ceftriaxone (16 mg/kg) intradermally, twice a day over a period of 5 days. Four mice were used as control (no antibiotic treatment-Day 0) and 5 mice were killd and tested by PCR and culture at each point of kinetics (Day1 to Day5) (29 mice total).

	Day 0		Day 1		Day 2		Day 3		Day 4		Day 5	
	Cult	PCR	Cult	PCR	Cult	PCR	Cult	PCR	Cult	PCR	Cult	PCR
Mouse 1	+	1.3	+	0.1	-	-	-	-	-	-	-	-
Mouse 2	+	1.3	+	0.06	-	-	-	-	-	-	-	-
Mouse 3	+	3.3	+	0.16	-	-	-	-	-	-	-	-
Mouse 4	+	1.2	+	0.11	-	-	-	-	-	-	-	-
Mouse 5			+	0.1	-	-	-	-	-	-	-	-
